# OP03 Regionalization And Patient-Centered Care: A Rapid-Realist Review And Implications For Health Technology Assessment

**DOI:** 10.1017/S0266462324000679

**Published:** 2025-01-07

**Authors:** Joan Quigley, Catherine O’Donnell, Neil Hawkins

## Abstract

**Introduction:**

Healthcare regionalization is the movement of responsibility of health care towards a regional body. It has been introduced in many countries and exists in many forms across high-, middle- and low-income countries. Supporters of regionalization purport that a more actively managed system, with better coordination and integration, could lead to improved quality and patient-centered care. However, evidence for this is unclear.

**Methods:**

Systematic searches combining terms for regionalization and patient-centered care in MEDLINE identified 5,765 titles for review. Three levels of screening were conducted by two independent reviewers: title only, abstract and title, and full-paper review. Rapid-realist synthesis methodology was used to gather a deeper understanding of the relationship between healthcare regionalization and patient-centered care, seeking to identify potential mechanisms and the context in which these operate. We also sought to determine whether novel methodologies such as this can be used by health technology assessment (HTA) bodies in an efficient manner that produces results directly applicable to decision-makers.

**Results:**

Studies from high-income countries, including Canada, New Zealand, Australia, and Italy, were included. The realist synthesis identified mechanisms by which whole healthcare-system regionalization can help or hinder the rollout of patient-centered care. Mechanisms were classified in relation to specific dimensions of patient-centered care including access and “patient as person.” Facilitators to the use of rapid-realist review in health policy include similarity of screening, searching, and extraction to traditional systematic review. Barriers include the scope of the literature considered relevant, length of time to familiarize with the method, and presentation of the findings in an accessible way for policymakers.

**Conclusions:**

This is the first realist synthesis of the relationship between whole healthcare-system regionalization and patient-centered care. Regionalization may help or hinder achievement of patient-centered care. Policymakers should note barriers to, and facilitators of, patient-centered care in the context of large-scale health system reform. Rapid-realist review has applications for HTA, particularly in the exploration of non-standard interventions.

